# Differences among [^18^F]FDG PET-derived parameters in lung cancer produced by three software packages

**DOI:** 10.1038/s41598-021-93436-w

**Published:** 2021-07-06

**Authors:** Agnieszka Bos-Liedke, Paulina Cegla, Krzysztof Matuszewski, Ewelina Konstanty, Adam Piotrowski, Magdalena Gross, Julian Malicki, Maciej Kozak

**Affiliations:** 1 Department of Macromolecular Physics, Adam Mickiewicz University, 61-614 Poznan, Poland; 2 Department of Nuclear Medicine, Greater Poland Cancer Centre, 61-866 Poznan, Poland; 3 Department of Medical Physics, Greater Poland Cancer Centre, 61-866 Poznan, Poland; 4 Chair, Department of Electroradiology, Poznan University of Medical Science, 61-701 Poznan, Poland

**Keywords:** Lung cancer, Non-small-cell lung cancer, Non-small-cell lung cancer, Imaging techniques

## Abstract

Investigation of differences in derived [^18^F]FDG PET metabolic and volumetric parameters among three different software programs in lung cancer. A retrospective analysis was performed on a group of 98 lung cancer patients who underwent a baseline [^18^F]FDG PET/CT study. To assess appropriate delineation methods, the NEMA phantom study was first performed using the following software: Philips EBW (Extended Brilliance Workstation), MIM Software and Rover. Based on this study, the best cut-off methods (dependent on tumour size) were selected, extracted and applied for lung cancer delineation. Several semiquantitative [^18^F]FDG parameters (SUV_max_, SUV_mean_, TLG and MTV) were assessed and compared among the three software programs. The parameters were assessed based on body weight (BW), lean body mass (LBM) and Bq/mL. Statistically significant differences were found in SUV_mean_ (LBM) between MIM Software and Rover (4.62 ± 2.15 vs 4.84 ± 1.20; *p* < 0.005), in SUV_mean_ (Bq/mL) between Rover and Philips EBW (21,852.30 ± 21,821.23 vs 19,274.81 ± 13,340.28; *p* < 0.005) and Rover and MIM Software (21,852.30 ± 21,821.23 vs 19,399.40 ± 10,051.30; *p* < 0.005), and in MTV between MIM Software and Philips EBW (19.87 ± 25.83 vs 78.82 ± 228.00; *p* = 0.0489). This study showed statistically significant differences in the estimation of semiquantitative parameters using three independent image analysis tools. These findings are important for performing further diagnostic and treatment procedures in lung cancer patients.

## Introduction

Lung cancer in general is one of the most commonly diagnosed (11.6%) types of cancer and is the leading cause of cancer-related death (18.4%) worldwide in both sex groups^1^, while non-small cell lung cancer (NSCLC) in particular is the most commonly (85%) newly diagnosed histopathological lung cancer type^2^. The diagnostic procedure involves establishing the diagnosis and staging the lung cancer. Apart from physical examination, imaging modalities, such as computed tomography (CT), endoscopic examination and positron emission tomography combined with computed tomography (PET/CT), play an important role in the diagnosis of lung cancer^3^. Combined PET/CT examination generally allows for a better assessment of the severity of the disease (including involvement of lymph nodes) than unimodal CT examination due to CT’s limited sensitivity (55%) for lymph node staging^4^. However, PET/CT has a higher (0.84–0.91) negative predictive value (NPV) in assessing the lymph nodes in T1 stage tumours, and when there is evidence of concomitant disease, such as sarcoidosis, tuberculosis or pneumoconiosis; therefore, endobronchial ultrasonography (EBUS) or oesophageal ultrasonography (EUS) is needed to confirm the malignant behaviour of suspicious lymph nodes^3, 4^.

[^18^F]Fluorodeoxyglucose (2-deoxy-2-[^18^F]fluoro-D-glucose, [^18^F]FDG) is the most commonly used radiotracer in PET/CT examinations. [^18^F]FDG PET/CT scans provide a number of metabolic and volumetric parameters, such as the maximum standardized uptake value (SUV_max_), mean standardized uptake value (SUV_mean_), metabolic tumour volume (MTV) and total lesion glycolysis (TLG). SUV is a normalized concentration of a radiopharmaceutical in a lesion of interest. Since both the patient’s body weight (BW) and lean body mass (LBM) can be used for normalization, both options should be examined alongside the nonnormalized uptake value (Bq/mL).

Imaging data and processing methodology are specific to each institution and have changed over the years due to the introduction of new techniques and software for PET/CT image interpretation.

In 2013, Sansone et al. performed a study in which the use of different software programs for SUV measurement was examined^5^. Three different software programs in only two patients were each analysed, and the results showed that the distribution of SUV differs among packages. However, the researchers did not alter the time-course analysis. Different approaches were shown by Arain et al., who compared four software packages and assessed the differences in various SUV values in 100 patients^6^. Their study concluded that although different software programs should not be used interchangeably in clinical practice, the differences in SUV values among them were small. Recently, Wilson et al. reviewed PET/CT images among four different FDA-approved software packages and found significant differences in SUV_max_ values among them^7^.

One of the most widely used packages of various applications is MIM Software (MIM Encore version 6.8.8, MIM Software Inc. Cleveland, OH, USA^8^). This tool is used in 3D [^18^F]FDG PET image analysis in a variety of medical areas, namely, oncological diagnostics and therapy, neurology and radionuclide dosimetry^9, 10^. In 2017, Breault et al. used this software in the analysis of [^18^F]florbetapir PET standard uptake value ratios (SUVr) in patients suspected of having Alzheimer’s disease^11^.

Phillips EBW (version 4.0.2.145, Philips Medical Systems Nederland B.V., the Netherlands^12^), implemented commercially with Phillips PET/CT scanners, is mainly used in oncology for diagnosis and treatment planning^13, 14^. Authors have also reported the value of this package in neurology^15^ and recently in COVID patients^16^.

Another software package applied for 3D quantitative image analysis of [^18^F]FDG PET data, also used in our study, is Rover (version 3.0.50, ABX GmbH, Radeberg, Germany^17, 18^). Torigian et al. reported the application of this tool in the diagnosis of a cohort of 15 patients with diffuse large B-cell lymphoma^19^. Furthermore, Segtnan et al. used this software to assess interobserver variability for processing [^18^F]FDG PET/CT studies of gliomas with regard to global hemispherical [^18^F]FDG uptake and cerebellar FDG uptake^20^. Recently, Mupparapu et al. found that Rover was suitable for quantification of the temporomandibular joint in patients with late-stage rheumatoid arthritis using [^18^F]FDG and [^18^F]NaF PET^21^.

The aim of this study was to compare values of [^18^F]FDG PET parameters obtained using three different commercially available software packages for PET image analysis (Philips EBW, MIM Software and Rover) in a group of previously untreated NSCLC patients. Such a comparison using the mentioned software has not been performed previously, especially on heterogeneous groups of patients. Moreover, the majority of papers compare only SUV values; thus, we decided to expand these findings by examining other PET/CT parameters that are routinely used in the clinic.

## Results

In the whole group of patients, a very strong correlation was found in SUV_max_ values in BW, Bq/mL and LBM among all three software programs and in SUV_mean_ BW and LBM, while a moderate correlation was found in SUV_mean_ values in Bq/mL among all three software programs (Tables 1 and 2). Another strong correlation was found between Rover and MIM Software in TLG (Bq/mL) value (r = 0.9863) and in MTV (BW) value (r = 0.9830) (Tables 3 and 4). Mean values for all assessed parameters in all three software programs are shown in Table 5.

**Table 1:** Pearson correlation coefficient for SUV_max_ values.

SUV_max_ correlation coefficient	BW	Bq/mL	LBM
Rover vs Philips	R = 0.9855	R = 0.9880	–
Rover vs MIM	R = 0.9991	R = 0.9859	R = 0.9561
Philips vs MIM	R = 0.9862	R = 0.9783	–

**Table 2:** Pearson correlation coefficient for SUV_mean_ values.

SUV_mean_ correlation coefficient	BW	Bq/mL	LBM
Rover vs Philips	R = 0.9328	R = 0.3053	–
Rover vs MIM	R = 0.9965	R = 0.4841	R = 0.9969
Philips vs MIM	R = 0.9330	R = 0.6394	–

**Table 3:** Pearson correlation coefficient for TLG value.

TLG correlation coefficient	BW	Bq/mL	LBM
Rover vs Philips	R = 0.0696	R = 0.0905	–
Rover vs MIM	R = 0.4524	R = 0.9863	R = 0.6014
Philips vs MIM	R = 0.2618	R = 0.1018	–

**Table 4:** Pearson correlation coefficient for MTV values.

MTV correlation coefficient	BW
Rover vs Philips	R = 0.1735
Rover vs MIM	R = 0.9830
Philips vs MIM	R = 0.1950

**Table 5:** Mean values and standard deviation (SD) for all assessed PET parameters from particular software.

Parameter	Philips EBW	MIM Software	Rover
SUV_max_ (BW)	9.95 ± 4.78	9.81 ± 4.70	9.89 ± 4.70
SUV_max_(Bq/mL)	31,220.72 ± 16,868.92	30,643.29 ± 17,042.99	30,864.33 ± 16,733.33
SUV_max_ (LBM)	–	7.30 ± 3.52	7.54 ± 3.63
SUV_mean_ (BW)	5.72 ± 2.64	6.15 ± 2.80	6.36 ± 2.86
SUV_mean_ (Bq/mL)	19,274.81 ± 13,340.28	19,399.40 ± 10,051.30	21,852.30 ± 21,821.23
SUV_mean_ (LBM)	–	4.62 ± 2.15	4.84 ± 1.20
TLG (BW)	349.83 ± 1053.30	146.28 ± 236.60	153.66 ± 242.03
TLG (Bq/mL)	2,009,159.10 ± 7,394,123.60	439,121.20 ± 698,372.40	464,196.30 ± 718,163.80
TLG (LBM)	–	111.09 ± 185.58	115.78 ± 190.63
MTV [cm^3^]	78.82 ± 228.00	19.87 ± 25.83	20.42 ± 26.05

Statistically significant differences were found in SUV_mean_ (LBM) between MIM Software and Rover (4.62 ± 2.15 vs 4.84 ± 1.20; *p* < 0.005), in SUV_mean_ (Bq/mL) between Rover and Philips EBW (21,852.30 ± 21,821.23 vs 19,274.81 ± 13,340.28; *p* < 0.005) and Rover and MIM Software (21,852.30 ± 21,821.23 vs 19,399.40 ± 10,051.30; *p* < 0.005), and in MTV between MIM Software and Philips EBW (19.87 ± 25.83 vs 78.82 ± 228.00; *p* = 0.0489).

No other statistically significant differences were shown in any other assessed parameters among the software programs.

## Discussion

In the present study, we examined the differences among [^18^F]FDG PET metabolic and volumetric parameters (SUV_max_, SUV_mean_, TLG and MTV) obtained from three software packages that are commercially available, namely, Philips EBW, MIM Software and Rover. The major finding included significant differences (*p* < 0.005) in SUV_mean_ (LBM) between MIM Software and Rover and in SUV_mean_ (Bq/mL) between Rover and Philips EBW (*p* < 0.005) and Rover and MIM Software (*p* < 0.005). Moreover, the MTV value showed significant differences (*p* = 0.0489) between MIM Software and Philips EBW. Additionally, strong correlations in SUV_max_ values (BW and Bq/mL, LBM) and SUV_mean_ (BW, LBM) among all three software packages were obtained. Furthermore, a strong correlation was found between Rover and MIM Software for MTV (BW) values (r = 0.9830) and TLG (Bq/mL) values (r = 0.9863).

SUV is a normalized concentration of a radiopharmaceutical in a lesion of interest. Since both the patient’s BW and LBM can be used for normalization, both options should be examined along with the nonnormalized uptake value (Bq/mL).

SUV_max_ is the maximal value among the voxels included in the region of interest (ROI), so it is completely independent of ROI definition but susceptible to noise^22^. Currently, because SUV_max_ is less dependent on the observer and is at the same time more reproducible, SUV_max_ is used more frequently than SUV_mean_^22–24^.

It was shown that different pathological types and sizes of NSCLC produce SUV_max_ values of different magnitudes in PET scans^25–27^. Moreover, the tumour differentiation of adenocarcinoma as well as the size of all NSCLCs can be impeccably predicted using SUV_max_ in [^18^F]FDG PET/CT, as demonstrated by Karam et al.^28^. Authors showed that a linear regression analysis of SUV_max_ from tumour size dependency could adequately distinguished adenocarcinoma from squamous cell carcinoma. Due to the small number of patients in our study, we did not distinguish NSCLC subtypes, so we cannot confirm or deny these findings. However, we do not exclude further analysis on a larger group of patients in the future. SUV_max_ strongly predicts not only lung cancer but also other types of cancers. It is important to mention multiple articles that focused on SUV_max_ [^18^F]FDG PET analysis in pretreated primary tumours for the prediction of the occurrence of neck metastasis in oral cancer^29^, head and neck squamous cell carcinoma^30^ and others^31, 32^. Despite strong evidence showing the scientific and medical value of SUV_max_, it should be noted that some other studies have revealed no correlation between SUV_max_ and tumour recurrence^33^.

The SUV_max_ values in our study were strongly correlated among each other in BW, Bq/mL and LBM in all three software programs, so it remained the most significant parameter for lung cancer prediction. SUV_max_ is based on a single voxel, which is the least observer- and ROI definition-dependent but strongly influenced by image noise^34^; therefore, this result should not be surprising. Nevertheless, this indicates that our work is significant for each study focusing on this type of prediction not only in lung cancer but also in other types of malignancies.

SUV_mean_ incorporates information from multiple voxels, making it highly dependent on voxels that are included in the analysis; thus, it is less sensitive to image noise^35^. In contrast to SUV_max_, it is rarely used as a metabolic biomarker, and only limited data exist to support it in this role. However, in several publications, the authors outlined the possible importance of SUV_mean_. It was shown, for instance, that an increase in the pretreatment SUV_mean_ of the primary tumour was associated with decreased disease-free survival (DFS)^36^. Moreover, a relative change in SUV_mean_ of more than 40% between baseline and after therapy was shown to differ by 2 years in overall survival, DFS and locoregional control^36^. Nevertheless, the authors outlined that SUV_max_ was a better predictor of disease outcome. It was also shown that SUV_mean_ assessed by [^18^F]FDG PET and supported by global hepatic glycolysis can reflect hepatic functional capacity. Authors have shown that this parameter can be used as a potential imaging diagnostic factor in assessing diffuse pathology of the liver^37^. Moreover, the relationship of both SUV_mean_ and SUV_max_ of [^18^F]FDG PET with an increasing number of metabolic syndrome components in visceral adipose tissue that are associated with vulnerability to atherosclerosis was recently described by Pakh et al.^38^. These are only a few recently reported possible applications of SUV_mean_.

According to the described data, nonnormalized SUV_mean_ values are highly differentiated among the three software programs used that raise questions about the consistency of the analysed parameters. Several studies have shown some inaccuracies in the SUV_mean_ value due to variations in ROI definition^22, 39^. To eliminate those inaccuracies, we used the average Th value over all software according to the specific size of the primary tumour.

There are a limited number of articles concerning SUV differences among available software packages. The most similar to our work is a study published in 2015 by Pierce et al.^40^ in which they showed substantial differences in SUV (BW) from a phantom study among tested PET/CT systems, which is in contrast to the results of this work. The authors would like to draw the readers’ attention to several limitations, such as investigation of single parameter (SUV (BW)), lack of clinical data (random noise) and small voxel size, that could have had a significant influence on the results of this project.

Investigations of changes in SUV values in patients were also presented by Brendle et al.^41^ and Hirji et al.^42^. Brendle et al. assessed the reproducibility of SUV values among different reconstruction methods (3D OSEM + TOF and PSF-reconstruction + TOF) and matrix sizes (3D OSEM: 200 × 200 and 400 × 400) in a cohort of 27 patients with different types of cancer. They found that SUV_max_, SUV_mean_ and SUV_peak_ do not differ significantly among themselves between different PET reconstruction methods. However, doubling the matrix size showed a tendency towards higher SUV values^41^. Hirji et al. analysed 25 patients to determine whether uptake in the aorta varies among different reconstruction algorithms. The homogeneity of the analysed group of patients was not defined. The differences in reconstructions between SUV_max_ or blood pool SUV_mean_ and target-to-background ratio were not statistically significant. However, qualitative analysis showed differences between IT + TOF and UHD or UHD + MAR reconstructions; therefore, harmonization of those techniques was recommended^42^.

In both works, the homogeneity of the patient group was questionable, and the number of analysed patients was relatively small. Conversely, the different histological tumour types may influence the analysed parameters. These limitations are in contrast to our study, in which all data were obtained on homogenous groups of NSCLC patients using the same reconstruction and the same matrix size to keep from influencing the PET parameters.

MTV together with TLG are volumetric PET parameters using a threshold-based volume of interest^43^. Since according to the current 8th TNM classification, tumour volume plays a crucial role in cancer staging, an analysis performed in a group of cancer patients is meaningful. Therefore, in our study, a phantom study was performed first, and the same threshold (dependent on the tumour size) was used in all three software programs.

Based on our analysis, we found a strong correlation in MTV among all three software programs. However, significant differences (*p* = 0.049) in this parameter were shown only between MIM Software and Philips EBW. Liu et al.^44^ showed that MTV differs significantly between patients with EGFR mutations and with wild-type EGFR. Shrestha et al.^45^ found that among all semiquantitative PET parameters, only MTV showed prognostic ability in patients with stage I NSCLC treated with carbon-ion radiotherapy. They also concluded that MTV histological variation may need consideration for risk-adapted therapeutic management^45^. Other authors suggest that MTV is a prognostic factor for local control (LC) and overall survival (OS) in patients with early-stage NSCLC^46^. MTV is also widely used for assessing gross tumour volume in radiotherapy planning not only in lung cancer patients but also in patients with other cancers^47^; therefore, based on our study, it is reasonable to assess patients on workstations with the same software to avoid under- or overestimating the results.

Currently, there is increased interest in TLG, which is a product of SUV_mean_ and MTV. It consists of both metabolic and volumetric information. Several studies have shown that TLG is a prognostic factor in lung cancer patients^48, 49^.

In our study, we found significant differences in SUV_mean_ and MTV between the assessed software programs; however, no differences were noted in TLG values, which might be caused by the small number of patients included in this analysis. The results of this work emphasize the requirement for meticulous analysis of depicted PET effects. One can see that the difference between [^18^F]FDG PET metabolic and volumetric parameters obtained in NSCLC (and not only) using different software programs can be crucial. Therefore, it should be emphasized that in pretreatment and even post-therapy analyses, parameters obtained from different software programs can be compared only qualitatively, and quantitative analyses should be harmonized^50, 51^. To the best of our knowledge, this is one of the very few studies that assess not only SUV values but also volumetric parameters (such as MTV and TLG) in a homogenous group of patients and not in a phantom study.

## Materials and Methods

### Patients

Retrospective analysis was performed on a group of 98 (42 F, 56 M) patients with NSCLC who underwent a [^18^F]FDG PET/CT study for initial staging after obtaining informed consent. All protocols were approved by the local bioethical committee (Bioethics Committee of Poznan University of Medical Science) as the retrospective analysis was based on standard examinations, and all research was performed in accordance with the Bioethics Committee guidelines and the Declaration of Helsinki.

### PET Acquisition

Acquisition was performed using a Gemini TF PET/CT scanner (OSEM reconstruction), 50–70 min after i.v. injection of [^18^F]FDG with a mean activity of 364 ± 75 MBq from the skull vertex to mid-thigh with a time per table of 1.30 min and slice thickness of 5 mm. Patients who had the examination performed more than 70 min after the injection were excluded from the analysis due to changes that occurred in the standardized uptake value (SUV). All patients fasted for at least 6 h before the examination (average glucose level was 102.91 ± 23.41 mg/dL). After administration of [^18^F]FDG, patients rested in a darkened room at room temperature. A simultaneous low-dose CT was performed.

The NEMA phantom study was used to identify the best cut-off method and corresponding optimal threshold value for primary tumour delineation in each software. The procedure was repeated for different tumour volumes represented by different sphere radii in the NEMA phantom. Afterwards, for each tumour volume, an average threshold over three software programs was calculated (Table 1). The averaged thresholds (Th) were used for all further evaluations in all three software programs. The purpose of the averaging procedure is to replicate standard clinical practice where the threshold values are taken from the literature and do not always represent the optimal value for a specific software.

### Evaluation

Several PET parameters (including SUV_max_, SUV_mean_, TLG and MTV) were cross checked by two independent observers and were obtained from three different software programs: Philips EBW (version 4.0.2.145, Philips Medical Systems Nederland B.V., the Netherlands^12^), MIM Software (MIM Encore version 6.8.8, MIM Software Inc. Cleveland, OH, USA^8^) and Rover (version 3.0.50, ABX GmbH, Radeberg, Germany^16^). SUV is defined as a ratio of tissue radioactive concentration [kBq/mL] at the time of injection and administered dose [MBq] at the time of injection and divided by the normalization factor. The normalization factor can be patient BW in kilograms [kg] (Eq. 1) or patient LBM in kilograms [kg] (Eq. 2). LBM relies on sex, height [cm] and body weight [kg] and is estimated using Eq. (3).^52^

1 $$SUV~(BW) = \frac{{measured~activity~concentration~[kBq/mL]}}{{administered~activity~[MBq] \times BW~[kg]}}$$

2 $$SUV~(LBM) = \frac{{measured~activity~concentration~[kBq/mL]}}{{administered~activity~[MBq]/LBM~[kg]}}$$

3 $$LBM = \left\{ {\begin{array}{*{20}l} {1.10 \times BW~[kg] - 128 \times \frac{{BW~[kg]^{2} }}{{height~[cm]^{2} }}} & {for~men} \\ {1.07 \times BW~[kg] - 148 \times \frac{{BW[kg]^{2} }}{{height~[cm]^{2} }}} & {for~women} \\ \end{array} } \right.$$

SUV in units of Becquerel’s per millilitre (Bq/mL) is nothing other than the measured uptake in the investigated ROI.

**Figure 1:**
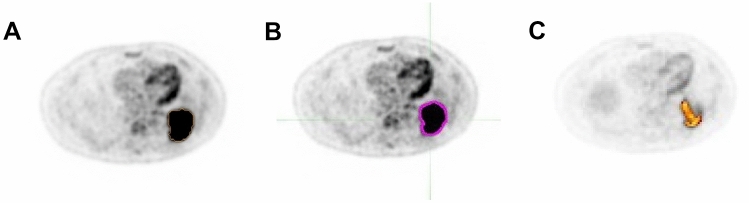
Delineation method performed on Philips EBW (**A**), MIM Software (**B**) and Rover (**C**).

All PET parameters were estimated using BW, LBM (except Philips EBW, which does not have this value in their workflow) and Bq/mL. The MTV was defined as the volume of the PET-positive tumour region. It was computed as the sum of the delineated tumour voxels (using an appropriate threshold Table 6) times the volume per voxel. An example of the delineation method based on one of the patients included in the analysis is shown in Fig. 1. Furthermore, TLG was calculated as a product of SUV_mean_ and MTV.

**Table 6:** Average cut-off Th based on NEMA phantom study.

Diameter of NEMA spheres [cm^3^]	MIM Software	Philips EBW	Rover	Average
1.150	Th65%	Th65%	Th62% & Th63%	Th64% (63.75)
2.572	Th57%	Th44% & Th45%	Th47% & Th48%	Th48%
5.575	Th48%	Th42%	Th42% & Th43%	Th44% (43.75%)
11.494	Th44%	Th44%	TH44%	Th44%
26.522	Th45%	Th44%	Th47%	Th45%

The normality of the data distribution was checked using the W Shapiro–Wilk test, and a *p*-value less than 0.05 was considered significant. Additionally, a Pearson correlation coefficient was used to compare parameters among software programs. A very strong correlation was assumed with values ranging from 0.7 to 1, a strong correlation ranging from 0.5 to 0.7, a moderate correlation ranging from 0.3 to 0.5 and a low correlation ranging from 0 to 0.3.

## Conclusions

The results of this work emphasize the requirement for meticulous analysis of depicted PET effects. The SUV_mean_ and MTV values showed the most significant differences among the assessed software programs. It should be noted that the difference between [^18^F]FDG PET metabolic and volumetric parameters obtained in NSCLC patients using different software programs might have an influence on further diagnostic and treatment procedures.
